# Dataset of N-doped CuO:NiO mixed oxide thin film sensor for glucose oxidation

**DOI:** 10.1016/j.dib.2020.106408

**Published:** 2020-10-14

**Authors:** Maghmood Palmer, Milua Masikini, Li-Wen Jiang, Jian-Jun Wang, Franscious Cummings, Mahabubur Chowdhury

**Affiliations:** a Department of Chemical Engineering, Cape Peninsula University of Technology, Bellvile 7535, South Africa; b State Key Laboratory of Crystal Materials, Shandong University, Jinan, Shandong 250100, PR China; c Electron Microscope Unit, University of the Western Cape, Bellvile 7535, South Africa

**Keywords:** Nitrogen doping, Mixed oxides, Glucose oxidation, Plasma etching, Copper oxide, Nickel oxide

## Abstract

In this data in brief article dataset of plasma-assisted nitrogen doping of a binderless, spin-coated CuO-NiO mixed oxide thin film was presented (Palmer et al., 2020). A comparison of the CuO, N–CuO/Cu_2_O, CuO:NiO and N–CuO/Cu_2_O:NiO are presented. The as prepared films were used for the application of a glucose sensor. The nitrogen doped species, generated during plasma ignition, resulted in a beneficial phase transformation of CuO to Cu_2_O. Characterisation techniques such as XPS, particle size distribution and EIS techniques were utilized to study the morphology, structural features, doping profile and electrical properties of the various developed electrodes. The electrochemical performance of the thin film sensors was tested using cyclic voltammetry and chronoamperometry. The CuO exhibited a sensitivity of 830 µA/mM cm^2^ up to 1.65 mM of glucose, N–CuO/Cu_2_O had a linear range up to 1.91 mM with a sensitivity of 873 µA/mM cm^2^ and the CuO:NiO electrode had a linear range up to 1.65 mM with a sensitivity of 1103 µA/mM.cm^2^ respectively. A detailed description of the methodology used is provided below.

## Specifications Table

**Table:** 

Subject	Materials Science – Metals and Alloys
Specific subject area	This article involves the synthesis of a thin film comprised of metal oxides and nitrogen composites for the application in glucose oxidation
Type of data	• Graphs • Figures
Acquisition of data	X-ray photoelectron spectroscopy (XPS): PHI X-tool, Ulvac-Phi XPS probe and analyzed using Origin Pro. Zeiss Auriga field emission scanning electron microscope. The particle size distribution was conducted using imageJ software. Autolab PGSTAT302N potentiostat (Metrohm, Switzerland) used for cyclic voltammetry, chronoamperometry and electrochemical impedance spectroscopy. Origin Pro used to analyse chronoamperometry and NOVA 2 software to fit equivalent electrical circuit.
Data format	• Raw • Analysed
Parameters for data collection	The XPS spectrograms are grouped according to the peaks achieved for the various elements. The CV experimentation is recorded at 25mV/s with an applied potential range of –0.1 to 0.8 V in a solution of 0.1 M NaOH. EIS data was recorded using a 0.01 V amplitude with a range of 1–10^6^ Hz. Chronoamperometric tests were conducted using the applied potential of the peak oxidation current.
Description of data collection	The electrodes were produced and sent for physical characterisation to Shandong University and University of Western Cape. Each electrode was tested electrochemically within the week of production. All raw electrochemical data was exported from NOVA 2 software and analysed using Origin Pro. After each electrochemical test, the electrodes (including counter and reference) were washed with deionised water and dried using compressed air blower. Washing of the beaker used for these tests were also done after each test and fresh electrolyte and analyte solutions were used.
Data source location	XPS was conducted using a PHI X-tool, Ulvac-Phi XPS probe at the State Key Laboratory of Crystal Materials, Shandong University, Jinan, Shandong 250100, PR China. SEM images were produced using a Zeiss Auriga field emission scanning electron microscope at the Electron microscope unit, University of the Western Cape, Bellvile-7535, South Africa. All electrochemical tests (CV, chronoamperometry and EIS) were done using an Autolab PGSTAT302N potentiostat at department of chemical engineering, Cape Peninsula University of Technology, Bellvile-7535, South Africa.
Data accessibility	Supplementary file entitled Raw data_DIB-D-20-01811 in .zip file format
Related research article	M. Palmer, M. Masikini, L. Jiang, J. Wang, F. Cummings, J. Chamier, O. Inyang and M. Chowdhury, Enhanced electrochemical glucose sensing performance of CuO:NiO mixed oxides thin film by plasma assisted nitrogen doping, Journal of Alloys and Compounds 853 (2021) 156900. https://doi.org/10.1016/j.jallcom.2020.156900

## Value of the Data

• Data provided in this manuscript provides an insight into the electronic and electrochemical properties of the N–CuO/Cu_2_O:NiO thin film electrode.
• Due to challenges in controlling the phase of deposited thin film, this data will be useful to researchers who are engaged in thin film deposition using various techniques.
• Other researchers around the world can use these data to conduct comparative studies on the effect of plasma assisted nitrogen doping in various other metal oxide thin films.

## 1 Data Description

### 1.1 Surface characterisation

**Fig. 1:**
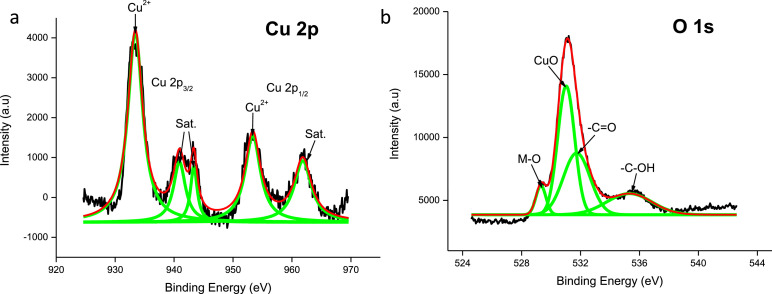
XPS data of the CuO electrode highlighting the (a) Cu 2p and (b) O 1s shells.

The raw data of the survey scan for the three electrodes described below can be found in the electronic supplementary file. XPS data of the pristine CuO electrode (Fig. 1) shows a Cu2p_3/2_ peak at 933.3 eV followed by the two satellite peaks at 940.9 and 943.4 eV. Moreover, it also shows a Cu2p_1/2_ peak at 953.3 eV and its satellite peak at 961.8 eV which matches the standard CuO XPS spectrum [1,2]. The oxygen binding energy of peaks 529.3 eV, which represents a common (metal) M–O bond [3,4], and 531.0 eV confirms the existence of oxygen in the CuO lattice and hydroxyl absorption which takes place on the electrode surface [2,5]. The O1s peak observed at 531.8 eV suggest that carbon contaminants are present on the electrode material in the form of a C=O bond [6].

**Fig. 2:**
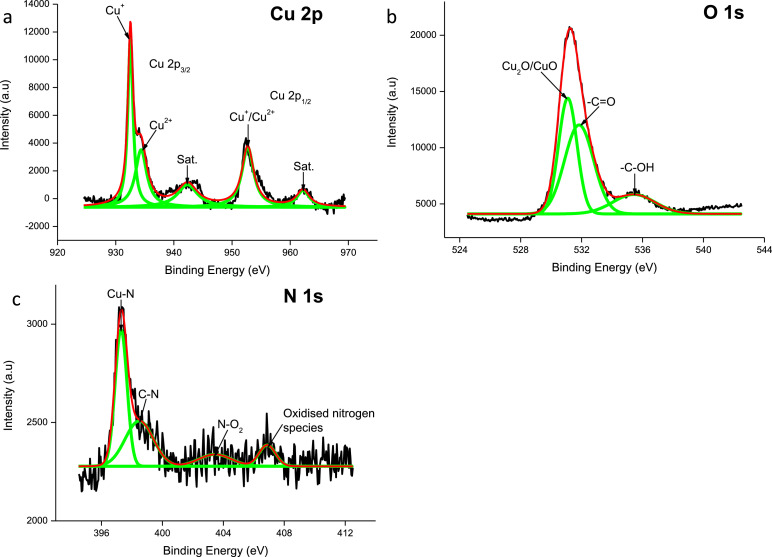
XPS data of plasma treated CuO film specifically focussing on the (a) Cu 2p, (b) O 1s and (c) N 1s orbital shells.

Cu(I) is present in the plasma treated CuO electrode due to the Cu2p_3/2_ peak observed at 932.5 eV and Cu2p_1/2_ peak at 952.7 eV [7,8]. The XPS data (Fig. 2) also shows a peak observed at 934.3 eV, indicating the presence of Cu(OH)_2_ [9]. The N1s spectrogram shows a clear presence of nitrogen within the plasma treated CuO electrode with a peak observed at 397.2 eV. This N1s peak indicated the existence of a M-N species in the form of pyridinic nitrogen [10], [11], [12]. A N1s peak of 398.5 eV hints to the existence of C–N [10]. Literature suggests that a N1s peak of 403.4 eV creates a nitrite bond of N–O_2_ with the host structure [13,14] and the binding energy of 406.8 eV suggest that other oxidised nitrogen species are present [15].

**Fig. 3:**
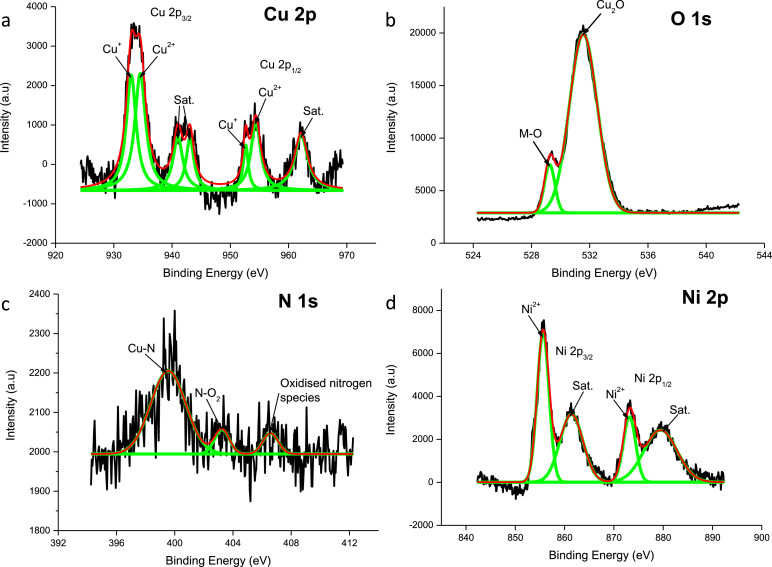
XPS data of N–CuO/Cu_2_O:NiO after electrochemical tests have been conducted, highlighting active peaks of the (a) Cu 2p, (b) O 1s, (c) N 1s and (d) Ni 2p shells.

A Ni 2p_3/2_ peak of 855.5 eV was observed (Fig. 3) for the as prepared electrode corresponding with a satellite peak at 861.2 eV suggesting that this thin film electrode has NiO within the active material [12,16]. Simultaneously, a Ni 2p_1/2_ peak at 873.2 eV and a satellite peak at 880.1 eV further confirm the existence of NiO. The corresponding Cu spectrum also shows the presence Cu_2_O, matching the bonds present in the plasma treated CuO electrode (Fig. 2). The binding energy of the O1s spectrum at 529.1 eV also confirms the existence of NiO [12,16] and 532.1 eV correspond to the formation of Cu_2_O [7,16] within the plasma treated CuO:NiO electrode. The N1s spectrum suggest that pyrrolic nitrogen is present due to the peak of 399.3 eV, which corresponds to a M–N bond [12]. The other peaks are consistent with the N1s spectrum observed for the plasma treated CuO electrode. XPS data of the N–CuO/Cu_2_O:NiO after electrochemical testing showed no changes in the spectrogram compared to virgin electrode (Fig. 3).

**Fig. 4:**
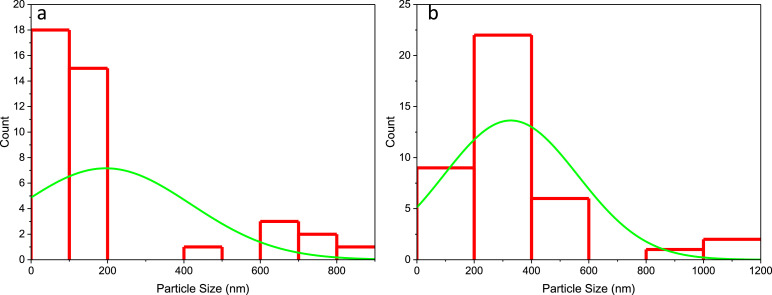
An average particle size distribution based on the SEM images of (a) CuO:NiO and (b) N–CuO/Cu_2_O:NiO electrodes.

The particle size distribution of the CuO:NiO electrode (Fig. 4(a)) shows an average particle size of less than 200 nm. Whereas, the distribution curve for the N–CuO/Cu_2_O:NiO electrode (Fig. 4(b)) exhibits a strong centralisation in particle size range of 200–400 nm.

### 1.2 Electrochemical behaviour

**Fig. 5:**
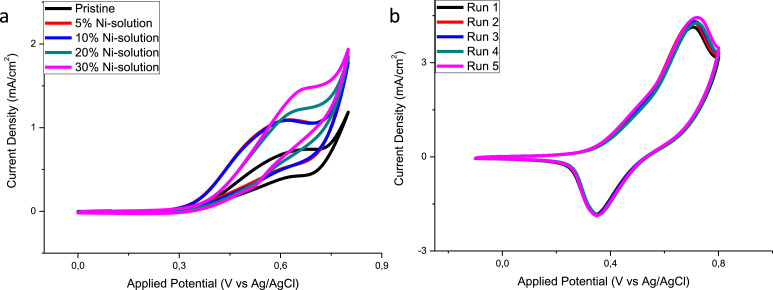
CV diagrams conducted at a scan rate of 25 mV/s for (a) comparison of the effects of Ni solution on the peak oxidation current and (b) repeatability of N–CuO/Cu_2_O:NiO sensor in 1 mM glucose solution.

All electrochemical raw data can be observed in the supplementary .zip file entitled “Raw data_DIB-D-20-01811”. Cyclic voltammetry (CV) experiments were conducted in alkaline solution of 0.1 M NaOH using a potential range from –0.1 V to 0.8 V vs Ag/AgCl at a scan rate of 25 mV/s in the presence and absence of glucose [3,17]. Electrodes prepared using the ratio of 70:30 (%V/V) copper to nickel precursor demonstrated the best electrochemical activity compared to the other ratios studied (Fig. 5(a)). The most effective electrode (N–CuO/Cu_2_O:NiO) was tested for repeatability in the presence of 1 mM glucose. The developed sensor exhibited an RSD of less than 3% for the repeatability (Fig. 5(b)).

**Fig. 6:**
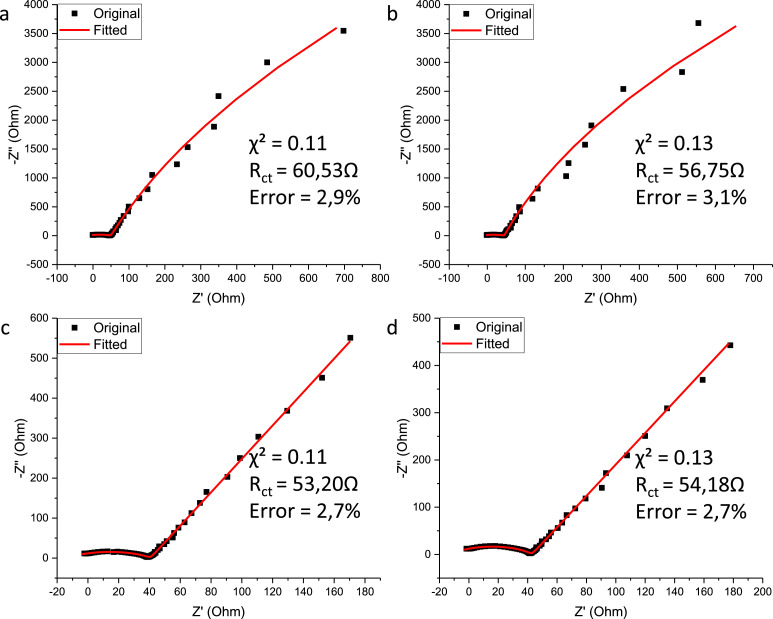
Fitted Nyquist plots of (a) CuO, (b) N–CuO/C_2_O, (c) CuO:NiO and (d) N–CuO/Cu_2_O:NiO electrodes.

The charge transfer resistance (*R_ct_*) quantifies the resistance to the flow of ions at the electrode and electrolyte interface [18]. The *R_ct_* values achieved from the modelled equivalent electrical circuit are presented in Fig. 6. Included in this figure is the χ², indicating how well the data fits the equivalent electrical circuit and the percentage error of the corresponding *R_ct_* value.

**Fig. 7:**
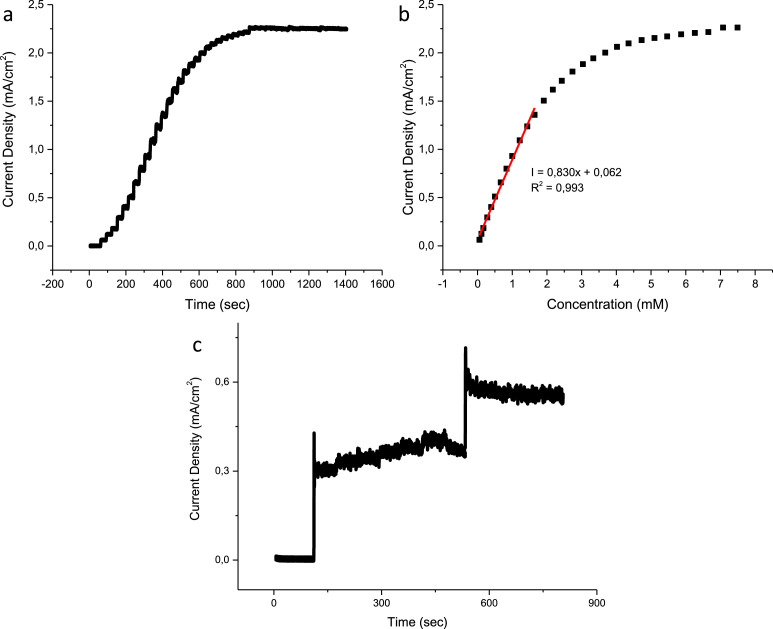
Amperometric response of the CuO sensor to (a) successive additions of glucose, (b) corresponding calibration curve including the linear range and (c) interference species of biological samples.

**Fig. 8:**
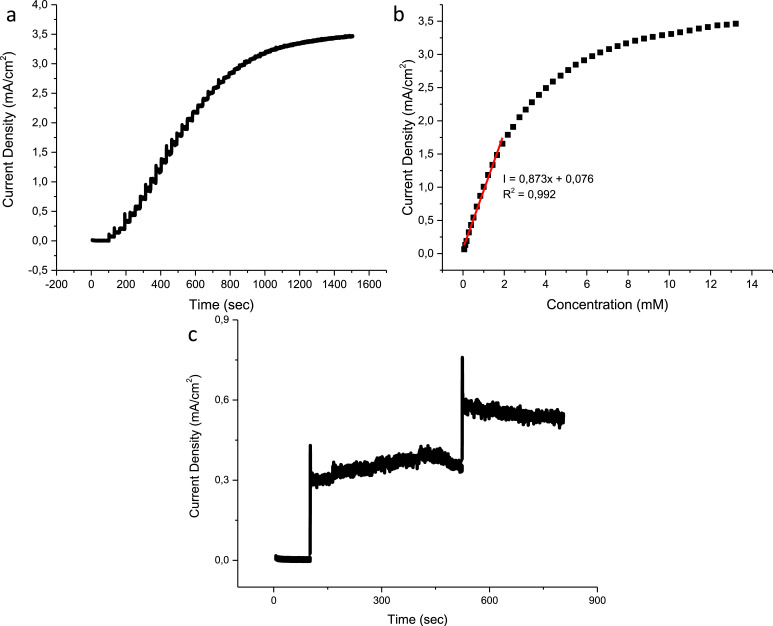
Amperometric response of the N–CuO/Cu_2_O sensor to (a) successive additions of glucose, (b) corresponding calibration curve including the linear range and (c) interference species of biological samples.

**Fig. 9:**
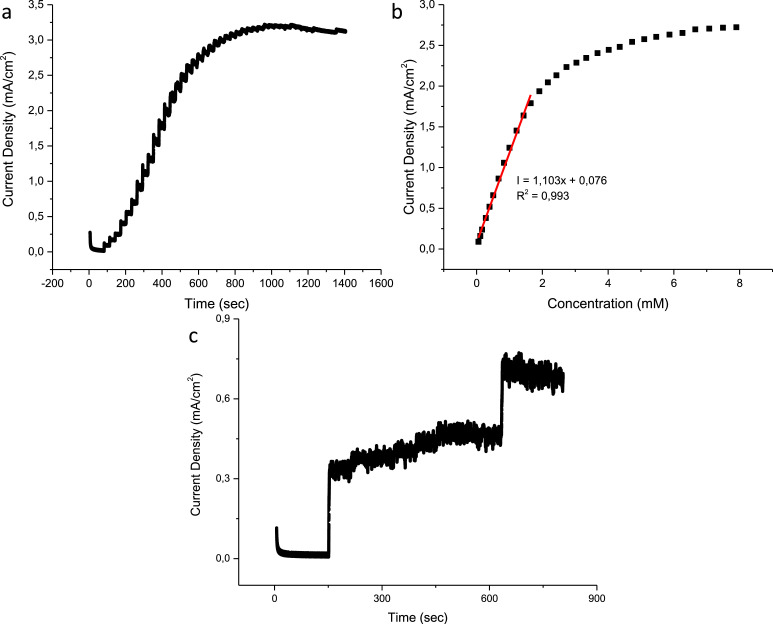
Amperometric response of the CuO:NiO sensor to (a) successive additions of glucose, (b) corresponding calibration curve including the linear range and (c) interference species of biological samples.

The chronoamperometric response of the CuO, N–CuO/Cu_2_O and CuO:NiO electrodes are presented in figures below including the interference tests. The linear range of the CuO electrode is found to be 1.65 mM with a sensitivity of 830 µA/mM cm^2^ (Fig. 7(b)). The N–CuO/Cu_2_O electrode has an improved linear range of up to 1.91 mM and a sensitivity of 873 µA/mM cm^2^ (Fig. 8(b)). A sensitivity of 1103 µA/mM cm^2^ was achieved by the CuO:NiO electrode for a linear range up to 1.65 mM of glucose (Fig. 9(b)). The selectivity of the electrode was performed in the presence of interference species i.e. uric acid, ascorbic acid, sucrose, and fructose. The developed sensors are selective towards glucose at the specified potential showing minimal interference with other species while maintaining a response to glucose after the successive additions of interference species.

## 2 Experimental Methodology

### 2.1 Materials

Copper (II) chloride dihydrate (CuCl_2_.2H_2_O), nickel (II) nitrate hexahydrate (Ni(NO_3_)_2_.6H_2_O), ethanol, hexane, toluene, sodium oleate (C_18_H_33_NaO_2_) Sodium hydroxide (NaOH), d-(+)-glucose, ascorbic acid (AA), uric acid (UA), sucrose, fructose, human serum and fluorine doped tin oxide (FTO) glass were purchased from Sigma Aldrich South Africa, deionised water and detergent were produced in-house by CPUT Bellville. All materials, besides FTO glass, were used without any further purification procedures.

### 2.2 Methodology of mixed oxide thin film electrode

Copper oleate was synthesised by ion exchange reaction between CuCl_2_.2H_2_O and C_18_H_33_NaO_2_ as reported in previous studies [19]. 20 mmol of CuCl_2_.2H_2_O, 60 mmol of C_18_H_33_NaO_2_, 40 ml of ethanol, 70 ml of hexane and 30 ml of deionised water were measured accurately and placed in a three neck round bottom flask. A condenser was connected to one of the openings, a thermometer was placed in another neck and the final neck was closed with a stopper. The setup was placed onto a heating mantel and a cooling system was connected to the condenser. The mixture was allowed to reflux at 70 °C for a total of four hours which allowed for the ion exchange reaction to take place. Thereafter, the mixture was washed a total of three times to remove all unnecessary by products. The final mixture was poured into a petri dish, covered with parafilm, and placed into a 60 °C oven overnight allowing for the excess moisture to be evaporated. This procedure created a wax like substance of copper oleate.

FTO glass slides were cut to size (1.2 × 4.5 cm) and extensively washed in an ultrasonic bath using detergent or degreaser for 10 min, ethanol bath for 10 min and finally in water for 10 min as well. The FTO glass slides were dried in a 60 °C oven over night following the cleaning procedure. Solution A of 0.09 g copper oleate was sonicated in 0.5 ml of toluene and solution B of 0.01 g Ni(NO_3_)_2_.6H_2_O was dispersed in 0.5 ml of ethanol. The nickel concentration was varied whereby solutions A and B were mixed thoroughly via ultrasonication in volumetric percentage ratios (70:30, 80:20, 90:10 and 95:5). A 50 µL aliquot of the precursor solution was deposited onto the cleaned FTO glass and spin coated in a twostep process to minimise edge/corner beads formation and to produce a uniform layer [20]. The FTO glass with precursor was spun at 1000 rpm for 10s followed by 4000 rpm for 50 s. Thereafter, the glass electrode was calcined at 350 °C for 10 min. The deposition, spin coating and calcination were repeated 1–6 times (each repetition is described as another layer forming onto the FTO glass slides) to achieve an optimum film thickness for improved electrochemical experiments.

### 2.3 Plasma assisted nitrogen doping of mixed oxide thin film

To obtain the nitrogen doping, the films were plasma irradiated in a custom-built plasma-enhanced chemical vapor deposition chamber at a base and treatment pressure of 1.5 × 10^−6^ and 7 × 10^−2^ mbar, respectively. Beforehand, the plasma power was opimised in a N_2_:Ar gas mixture by maintaining a constant gas flow rate of 100:100 sccm. An optimized plasma power of 500 W (plasma power supply = 5 V; 1.25 A) yield the most consistent and significant changes. Once set, the Ar flow was reduced to 0 sccm, which then yielded the red glowing N_2_ plasma. This point also signalled the start of the treatment, which was set at 10 mins for all samples. A summary of the electrode fabrication process can be found in [21].

### 2.4 Methodology of electrochemical tests

The electrochemical experiments were all conducted using an Autolab PGSTAT302N potentiostat (Metrohm, Switzerland). These experiments were done with a three-electrode setup: the as prepared electrodes as working electrodes, an Ag/AgCl reference electrode and a platinum wire counter electrode. A solution of 0.1 M NaOH was used as the electrolyte. The cyclic voltammograms were recorded in the potential range of –0.1 V to 0.8 V vs Ag/AgCl at 25 mV/s in a 0.1 M NaOH solution with various concentrations of glucose. The mixture of glucose in electrolyte was stirred prior to cyclic voltammetry tests. The raw data was recorded using NOVA 2 software and the corresponding graphs were plotted using Origin Pro.

Constant stirring conditions were applied for the chronoamperometry experiments using an applied bias potential achieved during CV test. This applied potential corresponds to the peak oxidation of current. Increasing concentrations of glucose was added to the electrolyte solution at 30 s intervals. The interference tests were conducted using the same chronoamperometric testing procedure. However, glucose (0.2 mM), ascorbic acid (0.02 mM), uric acid (0.02 mM), sucrose (0.02 mM), fructose (0.02 mM), human serum (0.02 mM) and glucose (0.2 mM) were added at 1 min intervals. NOVA 2 software was used to record all data and was analysed and plotted using Origin Pro.

EIS was determined at a bias potential of 0.3 V with 10 mV amplitude as the AC voltage in a frequency range between 1 Hz to 1000 MHz within a 0.1 M NaOH electrolyte solution. The EIS results are presented as Nyquist plots and is fitted using NOVA 2 software.

### 2.5 Surface characterisation

X-ray photoelectron spectroscopy (XPS) analysis was performed on a PHI X-tool fully automatic scanning microregion XPS probe (Ulvac-Phi) using monochromatic aluminum as the excitation source. The survey spectra were recorded with a pass energy of 160 eV and high-resolution spectra with a pass energy of 40 eV. The data recorded was analysed and plotted using Origin Pro. Zeiss Auriga field emission scanning electron microscope was used to study the morphology of the films produced. All thin films were imaged using an in-lens secondary electron detector with the beam accelerated to an electron high tension of 5kV. A particle size distribution of the thin film was analysed using imageJ software and plotted using Origin pro.

## Declaration of Competing Interest

The authors declare that they have no known competing financial interests or personal relationships which have, or could be perceived to have, influenced the work reported in this article.

## Appendix Supplementary materials

Image, application 1

## References

[1] Bai X., Chen W., Song Y., Zhang J., Ge R., Wei W., Jiao Z., Sun Y. (2017). Nickel-copper oxide nanowires for highly sensitive sensing of glucose. Appl. Surf. Sci..

[2] Li Z., Xin Y., Zhang Z., Wu H., Wang P. (2015). Rational design of binder-free noble metal/metal oxide arrays with nanocauliflower structure for wide linear range nonenzymatic glucose detection. Sci. Rep..

[3] Cheng S., DelaCruz S., Chen C., Tang Z., Shi T., Carraro C., Maboudian R. (2019). Hierarchical Co_3_O_4_/CuO nanorod array supported on carbon cloth for highly sensitive non-enzymatic glucose biosensing. Sens. Actuators B: Chem..

[4] Liu S., Hui K., Hui K. (2016). Flower-like copper cobaltite nanosheets on graphite paper as high-performance supercapacitor electrodes and enzymeless glucose sensors. ACS Appl. Mater. Interfaces.

[5] Dar M., Kim Y., Kim W., Sohn J., Shin H. (2008). Structural and magnetic properties of CuO nanoneedles synthesized by hydrothermal method. Appl. Surf. Sci..

[6] Dhara K., Ramachandran T., Nair B.G., Babu T.S. (2015). Single step synthesis of Au–CuO nanoparticles decorated reduced graphene oxide for high performance disposable nonenzymatic glucose sensor. J. Electroanal. Chem..

[7] Biesinger M.C. (2017). Advanced analysis of copper X‐ray photoelectron spectra. Surf. Interface Anal..

[8] Masudy-Panah S., Radhakrishnan K., Kumar A., Wong T.I., Yi R., Dalapati G.K. (2015). Optical bandgap widening and phase transformation of nitrogen doped cupric oxide. J. Appl. Phys..

[9] Biesinger M.C., Lau L.W., Gerson A.R., Smart R.S.C. (2010). Resolving surface chemical states in XPS analysis of first row transition metals, oxides and hydroxides: Sc, Ti, V, Cu and Zn. Appl. Surf. Sci..

[10] Wagner A.J., Wolfe G.M., Fairbrother D.H. (2003). Reactivity of vapor-deposited metal atoms with nitrogen-containing polymers and organic surfaces studied by in situ XPS. Appl. Surf. Sci..

[11] Soto G., De la Cruz W., Farıas M. (2004). XPS, AES, and EELS characterization of nitrogen-containing thin films. J. Electron. Spectrosc. Relat. Phenom..

[12] Zhu J., Yin H., Gong J., Al-Furjan M., Nie Q. (2018). In situ growth of Ni/NiO on N-doped carbon spheres with excellent electrocatalytic performance for non-enzymatic glucose detection. J. Alloys Compd..

[13] C. Wagner, A. Naumkin, A. Kraut-Vass, J. Allison, C. Powell, J. Rumble Jr, NIST X-ray photoelectron spectroscopy database, NIST standard reference database 20, version 3.4 (web version), U. S. Department of Commerce, (2003).

[14] Kabir S., Artyushkova K., Serov A., Kiefer B., Atanassov P. (2016). Binding energy shifts for nitrogen-containing graphene-based electrocatalysts–experiments and DFT calculations. Surf. Interface Anal..

[15] Matanovic I., Artyushkova K., Strand M.B., Dzara M.J., Pylypenko S., Atanassov P. (2016). Core level shifts of hydrogenated pyridinic and pyrrolic nitrogen in the nitrogen-containing graphene-based electrocatalysts: in-plane vs edge defects. J. Phys. Chem. C.

[16] Xiao X., Peng S., Wang C., Cheng D., Li N., Dong Y., Li Q., Wei D., Liu P., Xie Z. (2019). Metal/metal oxide@ carbon composites derived from bimetallic Cu/Ni-based MOF and their electrocatalytic performance for glucose sensing. J. Electroanal. Chem..

[17] Zhang Y., Liu Y., Su L., Zhang Z., Huo D., Hou C., Lei Y. (2014). CuO nanowires based sensitive and selective non-enzymatic glucose detection. Sens. Actuators B: Chem..

[18] Li X., Luo D., Zhang X., Zhang Z. (2015). Enhancement of electrochemical performances for LiFePO4/C with 3D-grape-bunch structure and selection of suitable equivalent circuit for fitting EIS results. J. Power Sources.

[19] Park J., An K., Hwang Y., Park J.-G., Noh H.-J., Kim J.-Y., Park J.-H., Hwang N.-M., Hyeon T. (2004). Ultra-large-scale syntheses of monodisperse nanocrystals. Nat. Mater..

[20] Chowdhury M., Ossinga C., Cummings F., Chamier J., Kebede M. (2017). Novel Sn doped Co_3_O_4_ thin film for nonenzymatic glucose bio-sensor and fuel cell. Electroanalysis.

[21] Palmer M., Masikini M., Jiang L.-W., Wang J.-J., Cummings F., Chamier J., Inyang O., Chowdhury M. (2020). Enhanced electrochemical glucose sensing performance of CuO: NiO mixed oxides thin film by plasma assisted nitrogen doping. J. Alloys Compd..

